# Efficacy of aggressive lipid-lowering therapy on systemic plaque stabilisation in acute myocardial infarction, assessed using non-obstructive general angioscopy

**DOI:** 10.1093/ehjcr/ytaf285

**Published:** 2025-06-17

**Authors:** Keisuke Kojima, Yasunari Ebuchi, Yasuo Okumura

**Affiliations:** Division of Cardiology, Department of Medicine, Nihon University School of Medicine, 30-1 Ohyaguchi-kamicho, Itabashi-ku, Tokyo 173-8610, Japan; Division of Cardiology, Department of Medicine, Nihon University School of Medicine, 30-1 Ohyaguchi-kamicho, Itabashi-ku, Tokyo 173-8610, Japan; Division of Cardiology, Department of Medicine, Nihon University School of Medicine, 30-1 Ohyaguchi-kamicho, Itabashi-ku, Tokyo 173-8610, Japan

Patients with acute myocardial infarction (AMI) are at high risk for systemic vascular complications, including ischaemic stroke and lower extremity artery disease, reflecting the widespread impact of atherosclerosis. Angioscopic evaluation has demonstrated that vulnerable plaques in the aorta contribute to major adverse cardiovascular events, underscoring the importance of early detection and management.^1^ While intensive lipid-lowering therapy is well established for coronary plaque regression, its effect on aortic plaques remains poorly understood.

Reports on pharmacologic strategies for stabilising aortic plaques are limited. To our knowledge, this is the first case to demonstrate aortic plaque stabilisation throughout the entire aorta using non-obstructive general angioscopy, providing angioscopic evidence of plaque regression following proprotein convertase subtilisin/kexin type 9 inhibitor therapy. A 50-year-old man with ST-elevation AMI underwent primary percutaneous coronary intervention (*Panels A* and *B*). Acute-phase angioscopy revealed an intense yellow plaque protruding at the stented site of the left anterior descending artery, along with multiple ruptured and thrombotic plaques throughout the aorta, suggesting extensive systemic atherosclerosis (*Panels D* and *F*; Supplementary material online, *Movie S1*). Despite high-intensity lipid-lowering therapy with rosuvastatin and ezetimibe, the LDL-cholesterol target of ≤55 mg/dL was not achieved. The addition of evolocumab successfully reduced LDL-cholesterol from 82 mg/dL to 21 mg/dL. Seven months later, follow-up angioscopy demonstrated that the previously observed intense yellow plaque in the coronary artery had stabilized (*Panels C* and *E*), while aortic angioscopy revealed a substantial reduction in ruptured plaques, thrombi, and yellow plaque intensity, indicating plaque stabilisation (*Panel F*; Supplementary material online, *Movie S2*). These findings prompted us to initiate a follow-up study to evaluate whether intensive lipid-lowering therapy can lead to sustained stabilisation or regression of aortic plaques. Global vascular intervention strategies, particularly intensive lipid-lowering therapy, are expected to contribute to the stabilisation of atherosclerotic plaques beyond the culprit lesion in patients with AMI.^2^ This case highlights the potential of pharmacologic therapy not only for coronary lesions but also for ruptured plaques in the aorta in patients with AMI.

**Figure:**
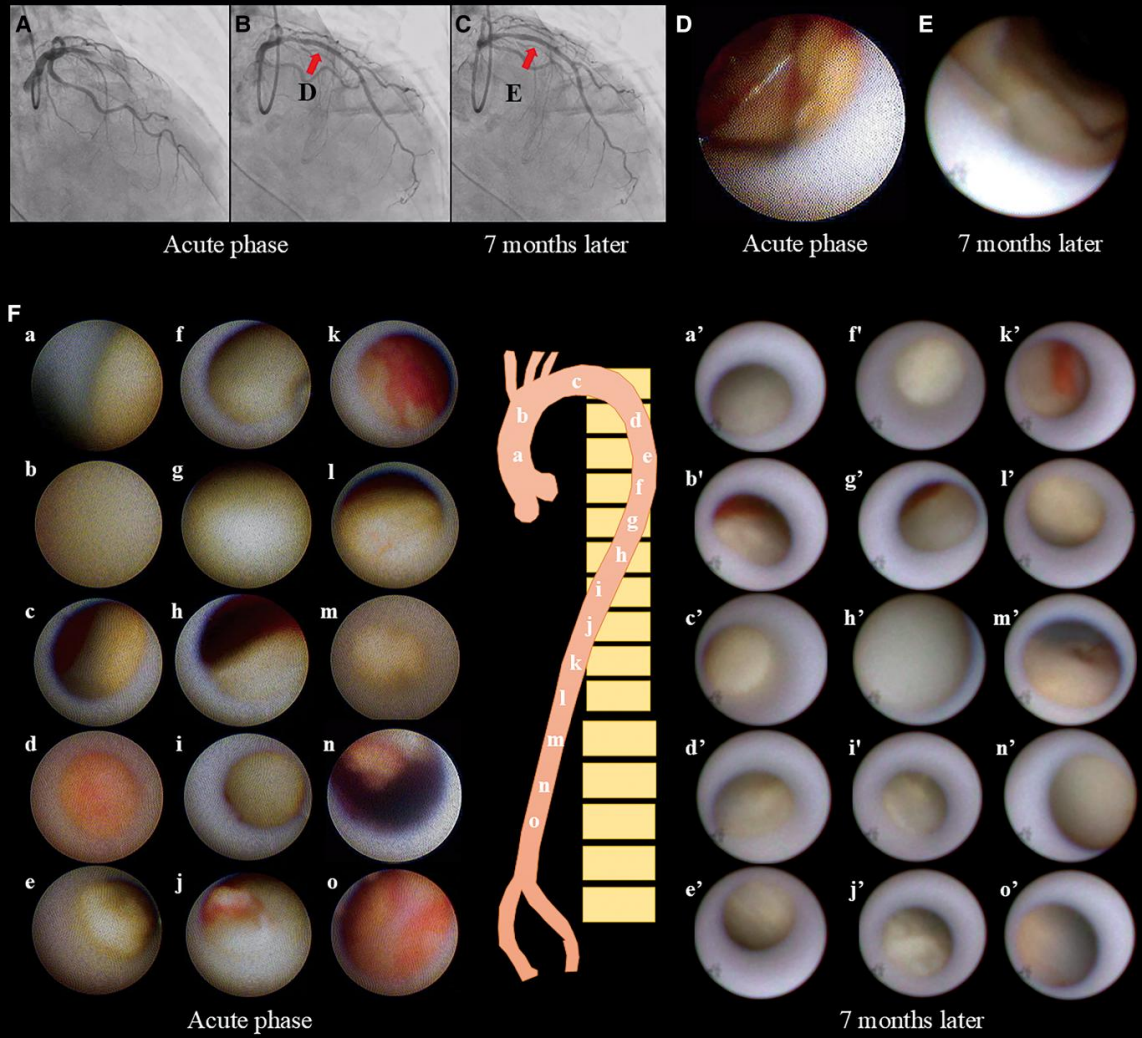


## Supplementary Material

ytaf285_Supplementary_Data

## Data Availability

The data underlying this article will be shared upon reasonable request to the corresponding author.

## Supplementary material

**Consent:** The authors confirm that they have obtained written consent from the patient for the submission and publication of this report, including images and associated text, in accordance with COPE guidelines.
